# Computational evaluation of selected compounds as potential inhibitors of gastric H⁺/K⁺-ATPase using molecular docking, molecular dynamics simulations, MMGBSA, and ADMET analyses

**DOI:** 10.1016/j.namjnl.2026.100105

**Published:** 2026-06-25

**Authors:** Emmanuel Israel Edache, Abdullahi Idi Mohammed, Hadiza Adamu Dawi, Aqel Albutti

**Affiliations:** a Department of Pure and Applied Chemistry, University of Maiduguri, Maiduguri, Borno State, Nigeria; b Department of Basic Health Sciences, College of Applied Medical Sciences, Qassim University, Saudi Arabia

**Keywords:** Gastric H⁺/K⁺-ATPase, Molecular docking, Molecular dynamics simulations, MMGBSA, ADMET, Anti-ulcer therapies

## Abstract

This study investigated the inhibitory potential of selected phytochemicals and conventional drugs against gastric H⁺/K⁺-ATPase, an important therapeutic target associated with acid-related gastrointestinal disorders such as peptic ulcers and gastroesophageal reflux disease. Computational approaches, including molecular docking, molecular dynamics simulations, MMGBSA free energy calculations, and ADMET profiling, were employed to evaluate the pharmacological relevance of the selected compounds. Molecular docking analysis revealed that several compounds exhibited favorable interactions with the target enzyme, with ciprofloxacin demonstrating stronger binding affinity than 3-hydroxypropyl oleate in both active-site and blind docking studies. Interaction analyses further showed that ciprofloxacin formed stable hydrogen bonds, hydrophobic interactions, and attractive charge interactions within the enzyme binding pocket. Molecular dynamics simulations confirmed the superior structural stability of the ciprofloxacin-protein complex through lower RMSD, RMSF, RoG, and SASA values compared with 3-hydroxypropyl oleate. Although MMGBSA calculations indicated slightly stronger total binding free energy for 3-hydroxypropyl oleate, ciprofloxacin displayed better overall compactness and interaction stability throughout the simulation period. ADMET and pharmoglyph analyses further established ciprofloxacin as the more promising candidate due to its favorable drug-likeness, high gastrointestinal absorption, acceptable solubility, lower metabolic risks, and excellent PharmoScore. The integrated computational findings suggest that ciprofloxacin possesses significant inhibitory potential against gastric H⁺/K⁺-ATPase and may serve as a promising scaffold for the development of safer and more effective anti-ulcer therapies.

## Introduction

Gastric H⁺/K⁺-ATPase, commonly referred to as the gastric proton pump, is a membrane-bound enzyme located in the parietal cells of the stomach lining (Lee et al., 2024). It plays a central role in gastric acid secretion by exchanging intracellular hydrogen ions (H⁺) for extracellular potassium ions (K⁺) using energy derived from ATP hydrolysis. This process is essential for maintaining the highly acidic environment of the stomach, which is necessary for digestion, activation of pepsin, and protection against ingested pathogens (McQuilken, 2021). The gastric H⁺/K⁺-ATPase belongs to the family of P-type ATPases and is composed of catalytic α- and glycoprotein β subunits that work together to regulate ion transport across the gastric membrane (Ramírez-Salinas et al., 2025). Overactivity or dysregulation of this enzyme is strongly associated with acid-related gastrointestinal disorders such as peptic ulcers, gastroesophageal reflux disease (GERD), Zollinger-Ellison syndrome, and gastritis (Smolka and Schubert, 2017). As a result, the enzyme has become an important therapeutic target in modern drug discovery. Proton pump inhibitors (PPIs), including omeprazole, lansoprazole, and pantoprazole, exert their pharmacological effects by irreversibly inhibiting gastric H⁺/K⁺-ATPase, thereby reducing gastric acid production and promoting ulcer healing (Adinortey and N’guessan, 2022; Miyazaki et al., 2018; Wołowiec et al., 2025). Due to its critical physiological and clinical importance, the gastric proton pump continues to attract significant interest in biochemical, pharmacological, and computational studies aimed at developing safer and more effective anti-ulcer therapies. Medicinal plants and naturally occurring chemicals are valuable sources of potential novel drugs. More specifically, because of their wide structural variety and low toxicity profiles, phytochemicals derived from plants are gaining interest as possible therapeutic agents against ulcers (Halder and Jha, 2023; Shahzad et al., 2024). These naturally occurring substances have an advantage over conventional medications in the treatment of ulcers, as they may target many pathogenic pathways (Yadav et al., 2024).

Computational methods are now crucial for researching and targeting gastric H⁺/K⁺-ATPase in drug discovery. In order to find interesting lead compounds before experimental confirmation, molecular docking is frequently used to anticipate the binding affinity and interaction patterns of putative inhibitors inside the active or allosteric regions of the proton pump (Edache et al., 2022a). These docking studies are often complemented by molecular dynamics (MD) simulations, which provide deeper insights into the stability, conformational flexibility, and time-dependent behavior of protein-ligand complexes under physiological conditions (Edache et al., 2022b; Ochu et al., 2017). Together, these techniques allow researchers to better understand inhibition mechanisms at the atomic level. In addition, ADME (Absorption, Distribution, Metabolism, and Excretion) profiling is applied to evaluate the pharmacokinetic properties and drug-likeness of candidate molecules targeting the proton pump (Ugbe et al., 2024). This ensures that potential inhibitors not only show strong binding affinity but also possess favorable bioavailability, safety, and metabolic stability. The integration of docking, molecular dynamics simulations, and ADME analysis, therefore, provides a powerful computational framework for the rational design and optimization of novel gastric H⁺/K⁺-ATPase inhibitors with improved therapeutic potential. Together, these computational approaches accelerate the rational design of safer, more effective acid-suppression therapies by providing detailed structural and dynamic insights that guide experimental validation.

## Materials and methods

**Fig. 1:**
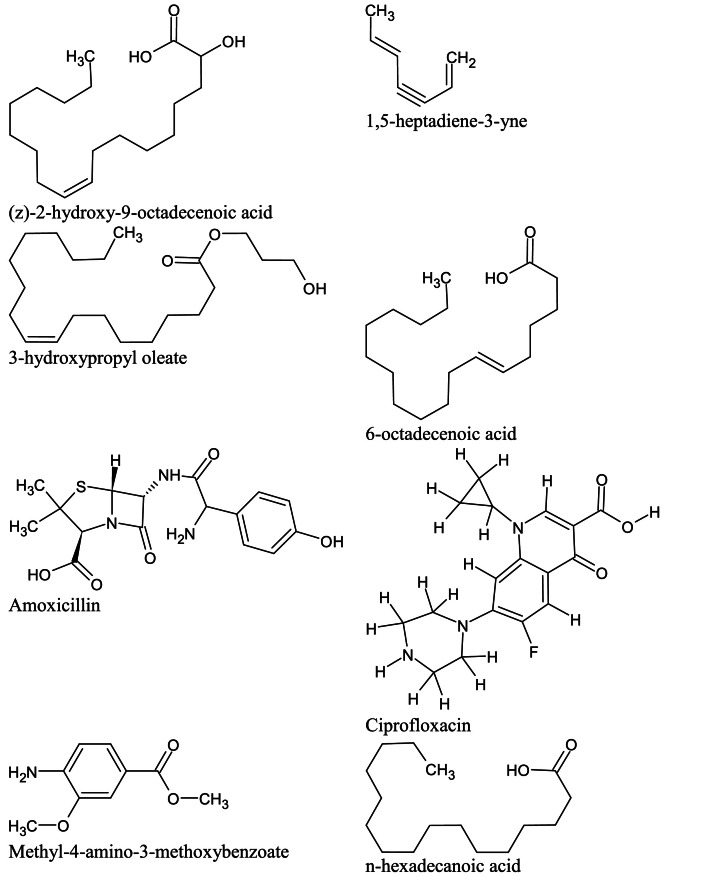

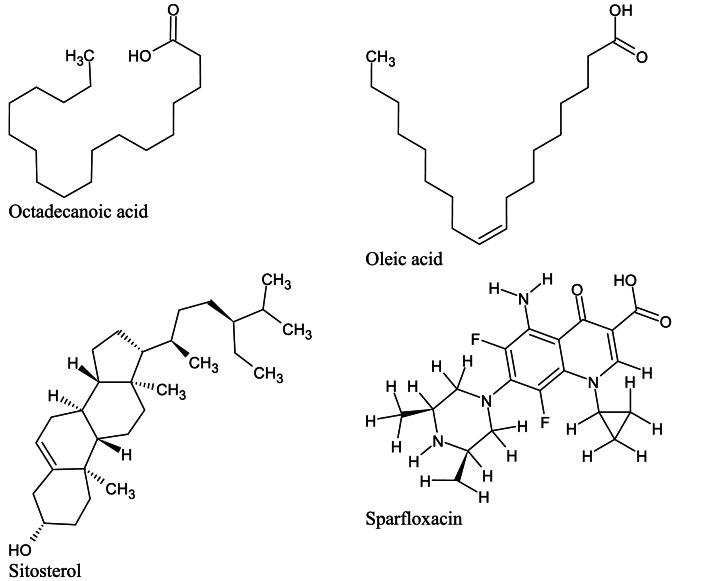
The physicochemical structures of the ten compounds and the reference drugs were obtained from the PubChem database in SDF format.

The smiles files of all the ligands are (z)-2‑hydroxy-9-octadecenoic acid, 1,5-heptadien-3‑yne, 3-hydroxypropyl oleate, 6-octadecenoic acid, oleic acid, octadecanoic acid, methyl-4-amino-3-methoxybenzoate, and n-hexadecanoic acid. Sitosterol, sparfloxacin, amoxicillin, and ciprofloxacin have been downloaded from the PubChem (https://pubchem.ncbi.nlm.nih.gov) database (Fig. 1) and were utilized for molecular modeling validations against ulcer therapies.

Following a preliminary conversion of the smiles file into SDF files of the phytochemicals using ChemDraw v16, Avogadro was used to transform each ligand into its three-dimensional (3D) structure (Hanwell et al., 2012). The MMFF94 force field was then used for geometry optimization to produce dependable and energetically stable conformations (Edache et al., 2023). In order to be ready for molecular docking investigations, the phytochemicals and a few chosen reference compounds were finally transformed into PDBQT format.

### Protein preparation

The three-dimensional (3D) structure of the gastric H⁺/K⁺-ATPase beta subunit (proton pump beta chain; PDB ID: 5YLU) was obtained from the Protein Data Bank. All heteroatoms were removed, and the protein structure was subsequently optimized using the protein preparation and minimization tools available in MzDock, an automated molecular docking pipeline (Kabier et al., 2024). The native ligand was first removed from the binding pocket, and the active site was defined based on the co-crystallized ligand (HKT1101). The grid box parameters for active-site docking were set to X = 49.023, Y = −16.387, and Z = −3.114, with dimensions of X = 12, Y = 16, and Z = 24 and a spacing of 0.375 Å for 5YLU. For blind docking, the coordinates were defined as X = 59.301, Y = −20.960, Z = 35.957, with grid dimensions of X = 94.591, Y = 104.749, Z = 146.954. The molecular docking simulations were performed using the EasyDockVina non-specific algorithm through the Vina Wizard implemented in AutoDock Vina v1.2.7 (Trott and Olson, 2010). The docked conformations were ranked according to binding energy, with the lowest energy value selected as the most favorable model (Edache et al., 2025a,b). Final pose selection was further validated by analyzing conventional hydrogen bonds and other key molecular interactions. Notably, lower binding energy values indicate stronger binding affinity. The docking results were visualized using Discovery Studio Visualizer.

### Molecular dynamics simulation (MDS)

Molecular dynamics simulation (MDS) is a thermodynamics-based technique used to investigate the dynamic behavior and structural changes of protein-ligand complexes over time (Edache et al., 2025a). In this study, MDS was performed on the selected protein-ligand complexes to evaluate the stability of the top-ranked ligands identified during the earlier screening stages. The simulations were carried out using the CHARMM36m force field and the TIP3P water model in NAMD v2.14 (Phillips et al., 2005). A 2-femtosecond time step with a multi-step integration algorithm was applied throughout the simulation. Protein-ligand complex PSF files were generated using the CHARMM-GUI web server, which also facilitated system solvation in a water box and neutralization with sodium (Na⁺) and chloride (Cl⁻) ions. Additionally, ligand topology and parameter files were prepared via CHARMM-GUI (Jo et al., 2009). Each system first underwent energy minimization for 20,000 steps before proceeding to a 4-nanosecond production run. The temperature was maintained at 310 K using a Langevin thermostat, and periodic boundary conditions were applied during the simulation. Visual Molecular Dynamics (VMD) was used for the last trajectory analysis and visualization (Humphrey et al., 1996). According to Edache et al. (2026), binding free energy calculations were performed using the Molecular Mechanics Generalized Born Surface Area (MM/GBSA) approach.

### ADMET prediction

In the pharmaceutical industry, prospective hit compounds' drug-likeness and ADMET profiles are crucial for minimizing side effects. The pharmoADMET technique was utilized in the current study to ascertain the effects of the physicochemical analysis (Thakur et al., 2026).

## Results and discussion

H⁺/K⁺-ATPase, the gastric proton pump, is a crucial enzyme that causes the production of stomach acid. One important treatment approach for treating acid-related conditions, including ulcers and gastritis, is to inhibit this enzyme. This work used molecular docking (active site docking and blind docking techniques) to assess the binding affinity of specific drugs against gastric H⁺/K⁺-ATPase.

All of the chosen compounds had negative binding energy against gastric H⁺/K⁺-ATPase, suggesting positive interactions with the target enzyme, according to a quick analysis of the binding affinity data (Table 1). When compared to other natural compounds, sitosterol showed the greatest active site binding affinity among the phytocompounds (−9.963 kcal/mol), indicating a very stable ligand-protein combination. Moderate binding interactions were shown by several fatty acid derivatives, including 6-octadecenoic acid (−6.125 kcal/mol) and 3-hydroxypropyl oleate (−6.244 kcal/mol). The lowest affinity, on the other hand, was shown by 1,5-heptadien-3‑yne (−4.408 kcal/mol), suggesting comparatively less stability within the binding region. Ciprofloxacin (−8.581 kcal/mol), sparfloxacin (−8.483 kcal/mol), and amoxicillin (−8.210 kcal/mol) showed significant binding energies at the active site that were either equal to or marginally lower than sitosterol when compared to conventional medications. Interestingly, amoxicillin demonstrated a highly constant and specific binding behavior between the active site (−8.210 kcal/mol) and blind docking (−8.217 kcal/mol). Overall, the findings show that sitosterol had the most promising binding affinity of all the phytocompounds that were evaluated, although the conventional medications also show robust and consistent interactions with the target enzyme.

**Table 1:** Binding Affinity of some selected compounds against gastric H(+)/K(+) ATPase.

S/N	Ligand	Active site affinity	Blind docking affinity
1	(z)-2‑hydroxy-9-octadecenoic acid	−5.538	−5.143
2	1,5-heptadiene-3-yne	−4.408	−4.269
3	3-hydroxypropyl oleate	−6.244	−4.771
4	6-octadecenoic acid	−6.125	−5.626
5	oleic acid	−5.980	−4.379
6	octadecanoic acid	−5.757	−4.996
7	methyl-4-amino-3-methoxybenzoate	−5.722	−5.455
8	n-hexadecanoic acid	−5.617	−5.105
9	Sitosterol	−9.963	−5.825
10	Sparfloxacin	−8.483	−7.928
11	Amoxicillin	−8.210	−8.217
12	Ciprofloxacin	−8.581	−7.113

Table 1 shows that ciprofloxacin has a much higher binding affinity for gastric H⁺/K⁺-ATPase than 3-hydroxypropyl oleate. Ciprofloxacin has a binding affinity of −8.581 kcal/mol in the active site, whereas 3-hydroxypropyl oleate has a binding affinity of −6.244 kcal/mol. Ciprofloxacin's greater negative value indicates a more robust and consistent contact with the catalytic region of the enzyme. Ciprofloxacin also records −7.113 kcal/mol in blind docking, which is stronger than the −4.771 kcal/mol found for 3-hydroxypropyl oleate. Ciprofloxacin may have improved overall binding stability over the protein surface, according to this. Ciprofloxacin has a larger potential inhibitory power against gastric H⁺/K⁺-ATPase than 3-hydroxypropyl oleate, as seen by its stronger binding affinity in both docking techniques.

**Fig. 2:**
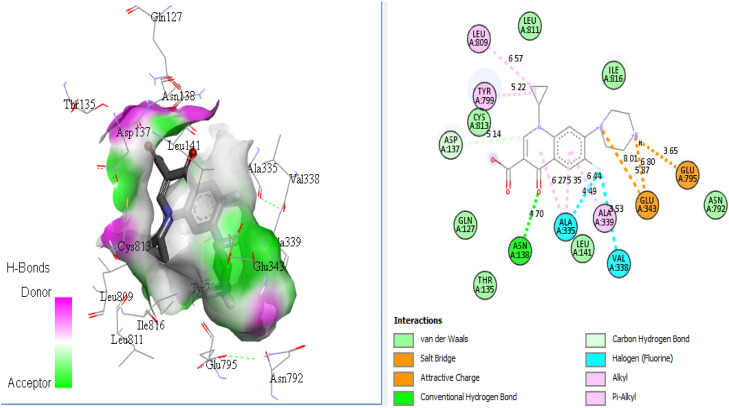
The protein-ligand interaction of ciprofloxacin with gastric H⁺/K⁺-ATPase protein.

Ciprofloxacin forms conventional and carbon-hydrogen bonds with the amino acids ASN138 and ASP137. As shown in Fig. 2, a variety of amino acids actively engage in the van der Waals interaction, including CYS813, ILE816, ASN792, LEU141, and many more. Additionally, a range of hydrophobic interactions between ciprofloxacin and amino acids was investigated, including pi-alkyl and alkyl. Fig. 2 illustrates the involvement of LEU809, TYR799, ALA335, and ALA339 in hydrophobic interactions. Nevertheless, GLU343 and GLU795 form an attractive charge interaction with the piperazine ring and a salt bridge. The halogen interaction (fluorine) involves the VAL338 and ALA335 molecules.

**Fig. 3:**
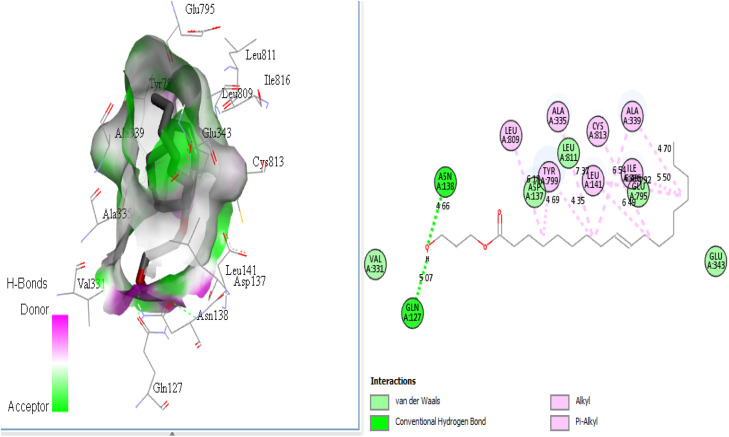
The protein-ligand interaction of ciprofloxacin with gastric H⁺/K⁺-ATPase protein.

Several amino acids, including VAL331, GLU343, and several others, are involved in the van der Waals interaction. Conventional hydrogen bonds are formed between the amino acids GLN127 and ASN138 by the 3-hydroxypropyl oleate. With the long chain of 3-hydroxypropyl oleate, the following compounds form hydrophobic interactions: LEU809, ALA335, CYS813, ALA339, TYR799, LEU141, and ILE816. However, as Fig. 3 illustrates, LEU809, ALA339, and TYR799 regularly interact with 3-hydroxypropyl oleate in alkyl and pi-alkyl ways.

## Molecular dynamics simulations

### Root mean square deviation (RMSD)

**Fig. 4:**
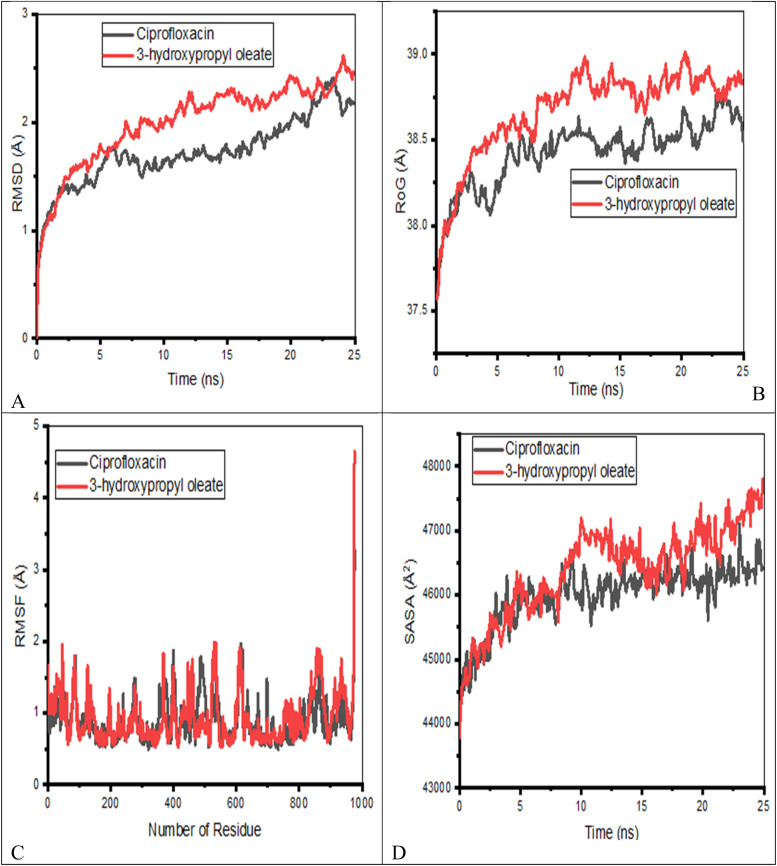
The molecular dynamics simulation plots of the gastric H(+)/K(+) ATPase complexed with ciprofloxacin and 3-hydroxypropyl oleate.

The Root Mean Square Deviation (RMSD) profile illustrates the variations in the structural stability of 3-hydroxypropyl oleate and ciprofloxacin throughout the course of the 25 ns simulation (Edache et al., 2024a; Ugbe et al., 2024b). Both compounds exhibit a sharp increase in RMSD at the start (0–2 ns), suggesting preliminary structural equilibration and adjustability within the binding environment (Fig. 4A). Although 3-hydroxypropyl oleate achieves somewhat higher values (∼1.4–1.5 Å), ciprofloxacin rises to around ∼1.3 Å. Ciprofloxacin exhibits mild fluctuations between 1.4 and 1.7 Å between about 3 and 10 ns, indicating rather steady binding with regulated structural flexibility. 3-hydroxypropyl oleate, on the other hand, has a progressive rising trend, beyond 1.8 Å and becoming close to ∼2.0 Å, suggesting a higher degree of structural variation. Ciprofloxacin increases steadily but more slowly after 10 ns until 25 ns, settling mostly between ∼1.8 and 2.3 Å. In contrast, 3-hydroxypropyl oleate shows significantly weaker stability and bigger conformational shifts, as seen by its higher RMSD values overall, which typically fall between ∼2.0 and 2.6 Å. Throughout the simulation, structural integrity is further supported by the lack of significant RMSF spikes. In comparison to 3-hydroxypropyl oleate (1.997 Å), ciprofloxacin has a reduced RMSD (1.720 Å), indicating a more stable protein–ligand combination with fewer structural variations during the simulation (Table 2). In contrast to 3-hydroxypropyl oleate, which exhibits larger deviations and more flexibility, ciprofloxacin generally displays lower RMSD values and more consistent variations throughout the simulation, indicating stronger structural stability in the system.

**Table 2:** The molecular dynamics simulation parameters of ciprofloxacin and 3-hydroxypropyl oleate.

Parameters	Ciprofloxacin	3-hydroxypropyl oleate
Root Mean Square Deviation (RMSD)	1.720	1.997
Radius of Gyration (RoG)	38.427	38.671
Root Mean Square Fluctuation (RMSF)	0.893	0.932
Solvent Accessible Surface Area (SASA)	45,992.433	46,390.926

### Radius of gyration (RoG)

The overall compactness of the molecular system over the 25 ns simulation is described by the radius of gyration (RoG) profile (Edache et al., 2024a,b). Ciprofloxacin and 3-hydroxypropyl oleate both exhibit a small rise in RoG from the start (0.1–2 ns), suggesting minimal structural relaxation and adjustment within the binding environment. While 3-hydroxypropyl oleate rises from ∼37.57 Å to ∼38.25 Å, ciprofloxacin climbs from ∼37.58 Å to ∼38.2 Å. Both systems oscillate within a limited range between about 2 and 15 ns (≈38.1–38.6 Å for ciprofloxacin and ≈38.2–38.9 Å for 3-hydroxypropyl oleate). Stable structural compactness without significant unfolding or collapse is suggested by these little oscillations. 3-hydroxypropyl oleate, on the other hand, continuously exhibits somewhat higher RoG values, suggesting a somewhat less compact structure. Ciprofloxacin stays largely between ∼38.4 and 38.8 Å with very little fluctuation between 15 and 25 ns. 3-hydroxypropyl oleate, on the other hand, exhibits much wider oscillations, sometimes coming close to ∼39.0 Å, indicating somewhat more structural flexibility or expansion (Fig. 4B). Ciprofloxacin and 3-hydroxypropyl oleate have relatively similar radii of gyration (RoG) values (38.427 Å and 38.671 Å, respectively), suggesting that the protein structures in both systems are similarly compact overall. Ciprofloxacin's somewhat lower RoG, however, points to somewhat improved structural compactness (Table 2). Overall, throughout the simulation, both systems retain a high degree of structural compactness. But compared to 3-hydroxypropyl oleate, which displays somewhat greater flexibility, ciprofloxacin has slightly lower and steadier RoG values, suggesting superior compactness and structural stability.

### Root mean square fluctuation (RMSF)

The flexibility of each amino acid residue during the course of the simulation is reflected in the root mean square fluctuation (RMSF) profile (Ugbe et al., 2024a,b). The protein backbone stays stable throughout the simulation, as seen by the generally low fluctuation values displayed by the majority of residues. The bulk of residues varies within a narrow range, indicating that the protein–ligand combination is stable and has little structural deformation. Some places do, however, exhibit greater RMSF peaks. Since loop regions and terminal residues are inherently more flexible than α-helices and β-sheets, these higher fluctuations are usually seen there (Fig. 4C). This kind of adaptability is normal and does not always mean that the system as a whole is unstable. The ligand-bound system, in contrast, preserves regulated residue variations, suggesting that ligand binding does not significantly destabilize the protein. Ciprofloxacin's RMSF value (0.893 Å) is also somewhat lower than 3-hydroxypropyl oleate's (0.932 Å), suggesting better structural stability and fewer residue-level variations (Table 2). Overall, the RMSF data indicate that the protein only exhibits limited flexibility in naturally dynamic areas and retains strong structural stability.

### Solvent accessible surface area (SASA)

The amount of the protein-ligand combination exposed to the solvent during the 25 ns simulation is described by the Solvent Accessible Surface Area (SASA) profile (Edache et al., 2022c). Ciprofloxacin and 3-hydroxypropyl oleate both exhibit an initial rise in SASA from about 43,800 Å² to approximately 45,000 Å² at the start (0–2 ns). This implies that when the system equilibrates in the solvent environment, it will undergo early structural correction and mild expansion. For both systems, SASA values vary considerably between 2 and 10 ns. In contrast to ciprofloxacin, 3-hydroxypropyl oleate starts to exhibit somewhat larger SASA values, sometimes surpassing ∼46,000–47,000 Å², suggesting increased solvent exposure. Ciprofloxacin maintains rather constant SASA values between 10 and 25 ns, with regulated variations, mostly between ∼45,800 and 46,800 Å². On the other hand, 3-hydroxypropyl oleate continuously shows greater SASA values, often rising to between 47,000 and 47,800 Å² as the simulation progresses (Fig. 4D). This suggests a little less compactness and more surface exposure. In comparison to 3-hydroxypropyl oleate (46,390.926 Å²), ciprofloxacin has a lower SASA (45,992.433 Å²), suggesting less solvent exposure and perhaps tighter binding inside the protein pocket (Table 2). Overall, neither system's solvent exposure varies much, and both systems continue to be rather stable. However, 3-hydroxypropyl oleate exhibits increased solvent accessibility and somewhat greater structural flexibility, while ciprofloxacin displays slightly lower and more stable SASA values, indicating superior structural compactness and tighter packing.

### MMGBSA of the selected ligands

The binding free energy and interaction contributions of 3-hydroxypropyl oleate and ciprofloxacin with the target protein are shown by the MMGBSA data (Table 3). There is no structural strain cost upon binding since both ligands exhibit negligible internal energy contribution (ΔE internal = 0). Ciprofloxacin has a greater positive value (18.49 kcal/mol) for the electrostatic plus solvation energy term (ΔE electrostatic + ΔG sol) than 3-hydroxypropyl oleate (8.68 kcal/mol). This implies that during binding, ciprofloxacin encounters a larger adverse polar/solvation contribution. On the other hand, both ligands have favorable (negative) van der Waals (ΔE VDW) interactions, suggesting that hydrophobic interactions play a major role in binding stability. Compared to 3-hydroxypropyl oleate (−23.32 kcal/mol), ciprofloxacin has a somewhat larger van der Waals contribution (−30.31 kcal/mol). Crucially, compared to ciprofloxacin (−11.82 ± 0.18 kcal/mol), 3-hydroxypropyl oleate has a greater negative total binding free energy (ΔG binding) at −14.64 ± 0.21 kcal/mol. 3-hydroxypropyl oleate shows superior expected binding strength in this system because increased binding affinity is indicated by larger negative ΔG values. In conclusion, MMGBSA analysis indicates that 3-hydroxypropyl oleate has a more favorable total binding free energy because of fewer unfavorable electrostatic/solvation penalties, although ciprofloxacin benefits from stronger van der Waals interactions. This suggests stronger and more stable binding.

**Table 3:** The free energy binding (MMGBSA) of the gastric H(+)/K(+) ATPase complexed with ciprofloxacin and 3-hydroxypropyl oleate.

Parameters	Ciprofloxacin	3-hydroxypropyl oleate
ΔE(internal)	0	0
delta E (electrostatic) + delta G (sol)	18.4914	8.678
delta E(VDW)	−30.3101	−23.3196
delta G binding (kcal/mol)	−11.8187 ± 0.1844	−14.6416 ± 0.207

### Pharmacokinetic and drug-likeness profile

Ciprofloxacin demonstrated a much stronger pharmacokinetic and drug-likeness profile, satisfying both Lipinski (Lipinski, 2016) and Veber criteria (Veber et al., 2002) with high GI absorption, acceptable solubility, low CYP interaction risk, and minimal plasma protein binding (Table 4). Its PharmoScore of 90/100 and high quantitative estimation of drug-likeness (QED) value suggest excellent oral drug potential (Bickerton et al., 2012), although the presence of an aniline toxicity alert indicates a possible safety concern that may require monitoring. In contrast, 3-hydroxypropyl oleate showed weaker ADMET behavior despite passing Lipinski rules marginally. Its high lipophilicity, poor solubility, excessive rotatable bonds, and Veber failure point to limited bioavailability and permeability challenges (Table 4). The compound also showed likely P-gp inhibition, high plasma protein binding, moderate CYP risks, and strong BBB permeability, which could increase the risk of CNS exposure and drug interactions. Although no structural toxicity alerts were detected, its low PharmoScore of 35/100 and poor overall drug-likeness make it a less favorable pharmaceutical candidate compared with ciprofloxacin (Table 4).

**Table 4:** Details of the pharmacokinetics drug-likeness profile of ciprofloxacin and 3-hydroxypropyl oleate.

Parameters	Ciprofloxacin	3-hydroxypropyl oleate
Molecular Weight	331.347	340.548
LogP	1.5833	5.9495
TPSA	74.57	46.53
HBD	2	1
HBA	5	3
Rotatable Bonds	3	18
Ring Count	4	0
Aromatic Ring Count	2	0
Fraction CsP₃	0.4118	0.8571
Heavy Atom Count	24	24
QED	0.8932	0.1904
ADME Behavior		
GI Absorption	High	Moderate
BBB Permeability	Moderate	High
Solubility	Good	Poor
LogS (estimated)	−2.0	−6.0
P-gp Substrate	Unlikely	Possible
P-gp Inhibitor	Unlikely	Likely
Caco-2 Permeability	High	High
Plasma Protein Binding	Low	High
CYP3A4 Risk	Low	Moderate
CYP2D6 Risk	Low	Moderate
Polarity	Low polarity	Low polarity
Confidence	High (100%)	High (100%)
PharmoScore	90/100	35/100
Toxicity Alerts		
Alert Count	1	0
Alerts	Aniline	None

### PharmoGlyph analysis

**Fig. 5:**
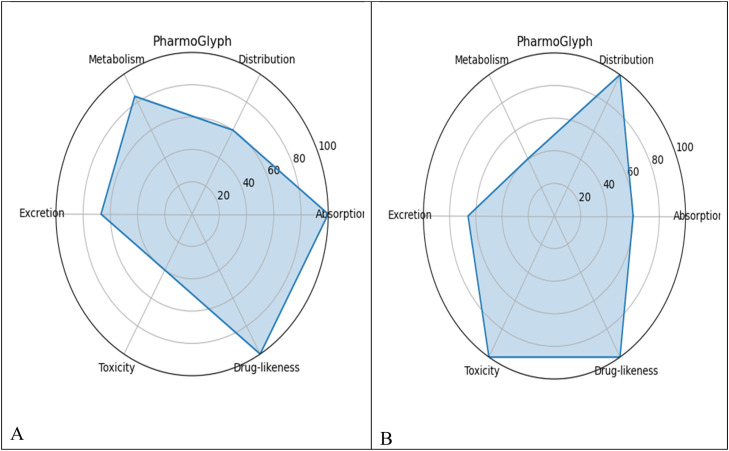
The pharmoGlyph analysis of (A) Ciprofloxacin and (B) 3-hydroxypropyl oleate.

The pharmacological behavior of 3-hydroxypropyl oleate and ciprofloxacin is visually compared using the pharmoglyph analysis. Ciprofloxacin's glyph represents a more favorable and balanced pharmacokinetic profile, which is consistent with the drug's strong drug-likeness, good absorption, adequate solubility, and reduced interaction concerns noted in the ADMET assessment (Fig. 5). Better applicability as a medicinal agent with dependable absorption and safer systemic behavior is suggested by its compact and optimized molecular properties. By contrast, 3-hydroxypropyl oleate's pharmacoglyph probably reflects a less optimal pharmacological profile with higher molecular flexibility, poor solubility, and high lipophilicity (Thakur et al., 2026). These characteristics are consistent with its lower PharmoScore and worse ADMET performance, suggesting potential difficulties with formulation, permeability balance, and metabolic interactions. Generally, the pharmoGlyph study confirms that 3-hydroxypropyl oleate may need structural improvement before being regarded as a promising therapeutic option, while ciprofloxacin has superior pharmaceutical potential.

## Conclusion

The current work effectively illustrated the value of integrated computational methods for assessing putative gastric H(+)/K(+) ATPase inhibitors. Ciprofloxacin showed more robust and stable interactions with the target enzyme than 3-hydroxypropyl oleate, according to a combination of molecular docking, molecular dynamics simulations, MMGBSA calculations, ADMET profiling, and pharmoGlyph studies. The molecule had acceptable pharmacokinetic properties, met major drug-likeness requirements, and maintained improved structural stability throughout the simulation, indicating its potential as a promising anti-ulcer treatment option. Although 3-hydroxypropyl oleate displayed a favorable binding free energy in MMGBSA analysis, its poor solubility, high lipophilicity, and weaker ADMET profile reduced its pharmaceutical suitability. These findings suggest that ciprofloxacin may provide a valuable structural framework for future gastric proton pump inhibitor development. Furthermore, the study emphasizes the importance of combining molecular docking, dynamics simulations, and pharmacokinetic evaluations in accelerating rational drug discovery and optimization processes for acid-related gastrointestinal disorders.

## CRediT authorship contribution statement

**Emmanuel Israel Edache:** Writing – review & editing, Writing – original draft, Visualization, Validation, Supervision, Software, Resources, Project administration, Methodology, Investigation, Funding acquisition, Formal analysis, Data curation, Conceptualization. **Abdullahi Idi Mohammed:** Writing – review & editing, Visualization, Resources, Investigation, Formal analysis, Conceptualization. **Hadiza Adamu Dawi:** Writing – review & editing, Supervision, Resources, Methodology, Funding acquisition, Formal analysis, Conceptualization. **Aqel Albutti:** Writing – review & editing, Writing – original draft, Validation, Supervision, Project administration, Methodology, Funding acquisition, Conceptualization.

## Declaration of competing interest

The authors declare that they have no known competing financial interests or personal relationships that could have appeared to influence the work reported in this paper.

## Data Availability

The data that support the findings of this study are available from the corresponding author upon reasonable request.
